# Prostaglandin A3 regulates the colony development of *Odontotermes formosanus* by reducing worker proportion

**DOI:** 10.1007/s44297-024-00030-3

**Published:** 2024-07-02

**Authors:** Qihuan Zhou, Ting Yu, Wuhan Li, Raghda Nasser, Nooney Chidwala, Jianchu Mo

**Affiliations:** 1 Ministry of Agriculture Key Lab of Molecular Biology of Crop Pathogens and Insect Pests, Key Laboratory of Biology of Crop Pathogens and Insects of Zhejiang Province, Institute of Insect Sciences, College of Agriculture and Biotechnology, Zhejiang University, Hangzhou, 310058 China; 2 Department of Zoology and Entomology, Faculty of Science, Minia University, El-Minia, 61519 Egypt

**Keywords:** *Odontotermes formosanus*, Prostaglandin A3, Population development, *Magnolia grandiflora* L., Metabolome analysis

## Abstract

Subterranean termites cause significant economic losses worldwide due to their destruction of agricultural and forest plants. In the past, soil termiticides were commonly used to control subterranean termites because they were effective and affordable. However, due to growing environmental concerns, these harmful substances have become less popular as they cause damage to non-target organisms and lead to environmental contamination. Baits crafted from plants and other easily metabolized compounds serve as excellent alternatives. In this study, we gathered branches from the promising plant, *Magnolia grandiflora* L. (MGL), along with branches from five other tree species that are potential food for termites. These branches were used as food to observe the population growth of *Odontotermes formosanus*. Additionally, a mix of branches from all six species was used to feed the control group (MIX). The study results showed that MGL nutrition significantly inhibited worker development, resulting in a significantly lower worker-to-soldier ratio (WSR). Furthermore, LC‒MS/MS analysis revealed that the level of prostaglandin A3 (PGA_3_) in workers significantly increased when they were under MGL nutrition. Additionally, ICP-MS analysis indicated a significant increase in calcium concentrations in the branches of MGL and combs under MGL nutrition. Moreover, there was a significant increase in peroxidase (POD) activity in workers under MGL nutrition. These findings suggest that the inhibitory effect of MGL nutrition on worker development may be due to excessive PGA_3_ synthesis, as Ca^2+^ and POD are involved in the synthesis process of PGs in insects. Subsequent verification experiments strongly support this hypothesis, as the WSR of colonies fed PGA_3_-added MIX was significantly lower than that of the MIX alone. This study introduces a new concept for developing environmentally friendly biological control methods for *O. formosanus* and sheds light on the potential role of PGs in termite development.

**Supplementary Information:**

The online version contains supplementary material available at 10.1007/s44297-024-00030-3.

## Introduction

Termites are known for their caste polyphenism and social lifestyle and are mainly found in tropical and subtropical regions worldwide [1–3]. Fungus-growing termites (Termitidae: Macrotermitinae) are a subgroup of higher termites known for their exclusive symbiosis with *Termitomyces*. This symbiosis not only allows for  lignocellulose degradation but also holds considerable edible and economic value [4, 5]. However, due to the feeding characteristics of termites, any plant containing lignocellulose can be their food [6].  Compared to lower termites, fungus-growing higher termites are larger in size, larger in population density, longer in feeding radius, more dominant in the ecosystem, and are more destructive to some extent [7]. Various species of fungus-growing termites are considered pests in Asia and Africa, causing extensive destruction to forest trees, crops, and wooden structures [2, 7]. They cause damage directly through gnawing on roots and stems and indirectly by transmitting pathogenic microorganisms [2, 8]. A diverse array of trees and crops, including eucalyptus, palm, coconut, mango, corn, wheat, coffee, peanut, cassava, tomato, cotton, and numerous other common agricultural and forestry crops, are involved in these termites’ diets [9, 10]. While not fully quantified, termites are estimated to cause economic losses of at least 40 billion USD annually worldwide, much of which are caused by fungus-growing termites [9, 11].

Various methods are employed for termite control, including physical barriers, chemical termiticides, and baits [11, 12]. Each method has its own advantages in different situations [7]. Physical barriers are well-used to protect small buildings, although they are expensive [13]. Chemical termiticides such as aldrin, dieldrin, fipronil, and thiamethoxam were once widely used for subterranean termite control because of their efficacy and cost-effectiveness [8, 10, 14]. However, these chemicals cannot control damage to non-targeted organisms and cause ecological risks [15, 16]. More flexible and environmentally friendly baits have become mainstream alternatives [11, 17, 18]. Baits combine food and toxicants and exploit foraging behavior to poison whole colonies [11, 17–19]. The selection of the toxicant determines the actual effect of the bait. Good toxicants need to have low-avoidance to foraging termites and be slow-release enough to spread the toxin to other nest mates by trophallaxis [19]. Since sugar-based arsenic baits were first proposed, various commercial baits have appeared in the last dozen years [11, 19, 20]. Most existing baits are designed against lower termites, mainly species in *Reticulitermes* and *Coptotermes* [7]. Chitin synthesis inhibitors (CSIs), such as hexaflumuron, noviflumuron, chlorfluazuron, and diflubenzuron, are slow-acting, dose-independent, and low-repellent and are widely recognized as good toxicants against Rhinotermitidae [8, 19]. However, these baits that achieve colony elimination by inhibiting molting are ineffective against fungus-growing higher termites, which do not need frequent molting [7]. Effective baits for higher termites are still lacking. The differences in multiple biological characteristics between fungus-growing termites and lower termites underscore the urgent need to develop new baits, especially against higher termites [21].

*O. formosanus* (Blattaria: Termitidae) is a higher subterranean termite widely distributed in Asia and Africa and poses a significant threat to trees [21–23]. *O. formosanus* displays a voracious appetite and can consume virtually any part of plants at any age, substantially harming diverse agricultural and forestry crops [2, 21, 23, 24]. In the summer of every year, alates of *O. formosanus* leaves their initial colonies and disperses to mate and establish new colonies [25]. With the continuous development of these newly established colonies, the growth of nearby plants is seriously jeopardized [26]. MGL is a popular landscape tree native to the coastal Southeastern United States and has been widely introduced in many countries worldwide [27, 28]. Termite channels can be observed on the branches of MGL in the overlapping distribution areas of MGL and *O. formosanus* in southern China. We stumbled upon an interesting phenomenon in the long-term study: branches of MGL seemed to be consumed faster than branches of other plant species collected from the same region in the laboratory culture of *O. formosanus*. However, in long-term observations in the field, we only found juvenile colonies less than two years old in the underground distribution of MGL, while mature colonies were found in the distribution areas of the other tree species used in the laboratory culture. Is this conflict between feeding preference and population development a coincidence, or is there an inherent regulatory mechanism?

In this study, we collected branches from MGL and five other tree species in a termite-infested region to use as food in order to assess the population growth of *O. formosanus* and investigate potential internal regulatory mechanisms. Unlike traditional termite control methods, the aim of this study is to manage the population of *O. formosanus* at the developmental level [18]. Our approach  emphasizes prevention and taking initiative than previous studies. Utilizing natural plants as bait for termite control is not only environmentally friendly but also avoids the drawbacks of traditional baits, such as high cost and labor intensity [24]. This study introduces a new concept for the research and development of new sustainable termite control baits. This research has considerable potential applications and significant research value, as it explores the role of PGs in termite physiology and reveals how PGA_3_ influences  termite population development.

## Materials and methods

### Sample collection and processing

#### Collection of termite colonies

Thirty-nine one-year-old well-growing fungal combs of *O. formosanus* were collected in two batches (21 for the feeding experiment and 18 for the verification experiment) underground in the wild area of Sanming City, Fujian Province, P. R. China (N 26° 23′, E 117° 61′). The major axis of each comb was between 6 and 7 cm, which ensured age proximity [29]. All combs were transported to the laboratory at Zhejiang University, Hangzhou City, Zhejiang Province (N 30° 18′, E 120° 5′) 48 h after excavation and were transferred to plastic culture boxes within 6 h after arrival. The cultivation conditions included darkness, a temperature maintained at 26 ± 2 °C, and internal humidity maintained at more than 80% by careful watering every week. Non-feeding cultivation was performed for two weeks until a termite channel appeared, after which specific food was added to each colony.

#### Food materials preparation

Fallen branches of six species of plant in the *O. formosanus* active area were collected at the campus of Zhejiang University. The plant species included MGL, *Cinnamomum camphora* (CCP), *Myrica rubra* (MRS), *Michelia figo* (MF), *Pinus elliottii* (PE), and *Osmanthus fragrans* (OFL). Branches of each species were put into an electric blast drying oven (Shanghai Boxun Industrial GZX-9070MBE) at 100 °C for more than 24 h separately until the weight was constant. All kinds of dried branches were stored in sealed plastic bags respectively. In the following experiments, the various kinds of foods (the dried branches) were named after the corresponding plant species: MGL, OFL, MF, PE, MRS, and CCP. The mixture of six kinds of foods was named MIX.

#### Termites cultivation

The feeding experiment consisted of seven groups with three replicates per group. A total of 21 colonies were involved. A total of 180 g of each kind of food was weighed and put into plastic bags to feed the six treatment groups, which were named after the food: MGL, OFL, MF, PE, MRS, and CCP. 30 g of each kind of food was  combined and mixed evenly in a plastic bag to feed the control group, which was named MIX. The control group contained six kinds of foods from which the termites could choose, simulating free and sufficient feeding conditions in nature. Each plastic bag was placed into the corresponding culture box for termite feeding. All colonies were kept under the same and suitable conditions described in "Collection of termite colonies" section (dark, 26 ± 2 °C, 80% RH) for 75 days. The comb establishment and colony reproduction status were analyzed after 75 days of cultivation.

The verification experiment consisted of six groups with three replicates per group, and a total of 18 colonies were involved. Each colony was fed 18 g of food, and some were mixed with specific chemicals. The experimental design and names of the six groups are shown in Table 1. For easy mixing with the chemicals, the foods used in this experiment were ground into powder using a high-speed grinder (Dongguan Fangtai Electric 800C). The methyl acetate solution of PGA_3_ obtained from the manufacturer (Shanghai Maokang Biotechnology, China) was blown under a low-speed nitrogen stream until the solvent was completely volatilized, and the PGA_3_ crystals were obtained. 1 mg of arachidonic acid (AA) (RHAWN, China) or PGA_3_ was dissolved in 10 mL of ethanol respectively and mixed well-mixed with 18 g MIX (3 g per kind of food) or MGL powder. The four obtained mixtures were placed in an 80 °C oven for 2 h until the ethanol was volatilized and the foods were dry. The remaining ethanol was blown down by a low-speed nitrogen stream until it volatilized completely. Another 18 g of MIX or MGL powder was mixed with 10 mL of ethanol and dried in the same way. Each kind of food was packed into 2 uncovered 50 mL centrifuge tubes and put into the corresponding culture box for the termites to feed on. All colonies were kept under the same suitable conditions described in "Collection of termite colonies" section (dark, 26 °C, 80% RH) for 30 days. The comb establishment and colony reproduction status were analyzed after 30 days of cultivation. Notably, to ensure the proper concentration of chemicals in the food, the amount of food consumed was changed to 18 g in the verification experiment, and the feeding duration was shortened to 1 month. A previous feeding experiment demonstrated that 18 g of food is sufficient for a colony to survive for one month.

**Table 1:** Group names and corresponding foods used in the verification experiment

Group name	MIX	MGL	MIXA	MGLA	MIXP	MGLP
Food composition	18 g MIX	18 g MGL	18 g MIX and 1 mg AA	18 g MGL and 1 mg AA	18 g MIX and 1 mg PGA_3_	18 g MGL and 1 mg PGA_3_

### Analysis of comb establishment and colony development of *O. formosanus*

#### Feeding rate calculation

The remaining food in each culture box was taken out from the plastic bag or centrifuge tubes. The soil mixed with food was picked out carefully, and the clean food was put into a 100 °C oven for 24 h until completely dry. The dry weight (M1) of the remaining food was then recorded. The initial weight of the food was recorded as M2. The feeding rate (FR) of each colony was calculated by the following formula:

$$\text{FR}= (\text{M}2-\text{M}1)/\text{M}2$$

#### Comb weight increment calculation

The initial weight (M3) of each comb was measured immediately after transfer to the laboratory. The final weight (M4) was measured after cultivation. The comb weight increment (CWI) of each comb was calculated by the following formula:

$$\text{CWI}=\text{M}4-\text{M}3$$

#### Analysis of population development status

All termites in each colony were carefully removed from the comb and soil using soft brushes and tiny tweezers. All clods were pulverized to ensure that no clods were missing. The numbers of workers and soldiers of each colony were counted and marked as worker: N1; soldier: N2. The WSR was calculated by the following formula:

$$\text{WSR}=\text{N}1 /\text{N}2$$

#### ICP-MS analysis

The contents of fourteen elements (Na, Mg, Al, Ca, V, Cr, Mn, Fe, Co, Ni, Cu, Zn, Ba, Pb) in the top layer comb and two kinds of foods (MIX and MGL) were analyzed by inductively coupled plasma‒mass spectrometry (ICP-MS) (PerkinElmer NexlON 300XX) with an octupole reaction system (ORS) (Yokogawa Analytical Systemes).

Pretreatment of the samples followed the methods of Toyama-Kato et al. [30] with some modifications. A total of 0.1 g of top layer comb or food powder was collected and placed in a PTFE beaker, and 1 mL of concentrated nitric acid (65% ~ 68%) was added. Each beaker was sealed with sealing film and then placed on a 60 °C heating tray (Hangzhou Youning Instrument HS-350C) for 4 h, after which 1 mL of concentrated nitric acid was added, followed by stepwise heating at 75 °C for 0.5 h, 130 °C for 0.5 h, and finally 200 °C for 0.5 h. The solution was filtered through an aqueous filter membrane with a pore size of 0.22 μm. Then, 0.75 mL of filtrate was added to a 25 mL volumetric bottle, which was then filled with deionized water to 25 mL, and a 3% diluted solution was obtained. The follow-up test was completed by the Physical and Chemical Analysis Room of the Department of Agricultural Biological Environment, Zhejiang University.

#### Analysis of POD activity in workers

A POD activity test kit (Jiangsu Addison Biotechnology, China) was used to measure POD activity. Ten workers from each colony were weighed (W) and ground into a homogenate together with 1 mL of extraction liquid. The homogenate was centrifuged at 12,000 × g for 10 min at 4 ℃ in a centrifuge (Eppendorf 5810R). Then, 160 µl of tissue supernatant, 160 µl of reagent 1, 440 µl of reagent 2, and 40 µl of reagent 3 were successively added to a 1 ml glass cuvette and put into a spectrophotometer (Thermo Fisher Scientific NanoDrop2000) immediately after mixing well to measure the absorption at a wavelength of 470 nm. The above reaction was carried out at 25 °C, and the reaction started immediately after reagent 3 was added. The light absorption values at 20 s (A1) and 80 s (A2) were recorded. The reaction time (T) was 1 min. One POD activity unit (U) was defined as an increase in the absorption at 470 nm of 0.5 per g tissue per minute. The formula for calculating POD activity was as follows:

$$\text{U}=12.5 \times (\text{A}2-\text{A}1) /\text{T}/\text{W}$$

#### Termite metabolite analysis under MGL nutrition

Non-targeted metabolomics analysis of workers and queens under MGL and MIX nutrition (named MGL-Q, MGL-W, MIX-Q, MIX-W, four replicates for MGL-Q, and three replicates for the other groups) were analyzed based on liquid chromatography‒mass spectrometry (LC‒MS/MS) technology [31, 32]. One queen or ten workers were collected for each replicate and stored at -80 ℃ before analysis. Two queens were found in colony 1 of the MIX. To ensure the integrity and reliability of the data, these two queens were tested together with the two queens in colonies 2 and 3 of the MIX. MIX-Q contained four replicates. LC‒MS/MS analysis was performed using a Vanquish Ultra High-Pressure Liquid Chromatography (UHPLC) system (Thermo Fisher Scientific) coupled with an Orbitrap Q ExactiveTMHF-X mass spectrometer (Thermo Fisher Scientific) at Novogene Co., Ltd. (Beijing, China).

### Statistical analysis

The raw data files generated by UHPLC-MS/MS were processed using Compound Discoverer 3.1 (Thermo Fisher Scientific) to perform peak alignment, peak picking, and quantitation for each metabolite. The metabolomics data processing software metaX was used to transform the data, and then principal component analysis (PCA) and partial least square discriminant analysis (PLS-DA) were performed to obtain the VIP value of each metabolite. Statistical analyses for this part were performed using the statistical software R (R version R-3.4.3), Python (Python 2.7.6 version), and CentOS (CentOS release 6.6). Univariate analysis (t-test) was applied to calculate the statistical significance (*P*-value), and the fold change value (FC) of metabolites between the two groups was calculated. The metabolites with VIP > 1 and *P*-value < 0.05 and fold change ≥ 2 or FC ≤ 0.5 were considered to be differential metabolites. The functions of these metabolites and the metabolic pathways were studied using the KEGG database (https://www.genome.jp/kegg/pathway.html). “N” is the number of metabolites involved in the KEGG metabolic pathway among all metabolites, “n” is the number of differential metabolites in “N”, “y” is the number of metabolites annotated to a certain KEGG pathway, and “x” is the number of differential metabolites enriched to that KEGG pathway. The metabolic pathways enrichment of differential metabolites was performed, when the ratio was satisfied by x/n > y/N, the metabolic pathway was considered to be enriched, and when the *P*-value of the metabolic pathway was < 0.05, the metabolic pathway was considered to be significantly enriched.

Statistical analyses for the other parts were performed using SPSS (V21.0). Independent Samples Test and one-way ANOVA were used for statistical analysis. Significance levels are indicated as *P* < 0.05 (*), *P* < 0.01 (**), or *P* < 0.001 (***). In multiple comparisons, different letters indicate different statistical groupings and two completely different sets of letters indicate significant differences between the corresponding groups.

## Results

### Comb and population development of *O. formosanus* under different nutrient conditions

**Fig. 1:**
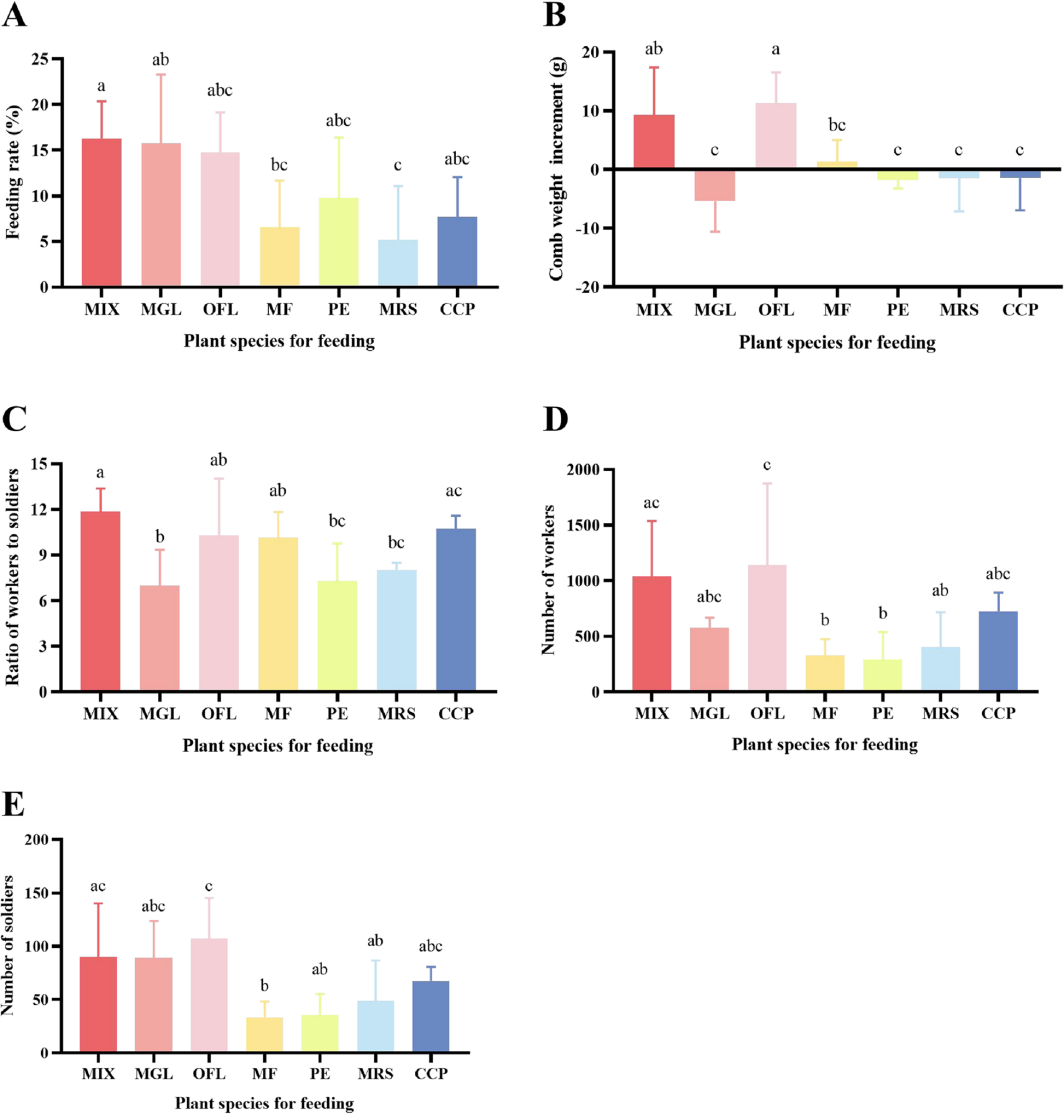
Growth status of *O. formosanus* under different nutrient conditions. Different letters above the bars indicate different statistical groups. Two sets of letters that do not overlap indicate a significant difference between the two corresponding treatments; there is no significant difference between the two treatments that are included in any of the same statistical groups (*P* < 0.05, one-way ANOVA). **A** Feeding rates of termites under different nutrient conditions. **B** Comb weight increments under different nutritional conditions. **C** Ratio of workers to soldiers under different nutritional conditions. **D** Number of workers receiving different nutrients. **E** Number of soldiers under different nutrition conditions

Figure 1A shows that the FRs of all the treatments were lower than that of the control. The preferences of workers for the six kinds of single foods were ranked as follows: MGL > OFL > PE > CCP > MF > MRS (Fig. 1A). The FRs of MF and MRS (6.54% and 5.17%, respectively) were the lowest, as the former was significantly lower than that of MIX (16.30%), while the latter was significantly lower than that of MIX and MGL (Fig. 1A). Notably, the FR of MGL (15.72%) was the highest among the six treatments, almost on par with the MIX, while the CWI under MGL nutrition (-5.35 g) was negative (Fig. 1A and B). In addition, PE (-1.84 g), MRS (-1.46 g) and CCP (-1.42 g) also caused varying degrees of comb weight reduction after 75 days of culture (Fig. 1B). The CWIs under the seven nutrient treatments were ranked from highest to lowest as OFL > MIX > MF > CCP > MRS > PE > MGL (Fig. 1B). The MGL had the lowest CWI, which was significantly different from that of the MIX (9.29 g; Fig. 1B). The poor state of comb development under MGL nutrition was caused by inappropriate nutrient intake. In addition to the development of comb, the WSR of the MGL (6.99) was also the lowest among the seven groups (Fig. 1C). The WSRs of the seven groups were ranked as MIX > OFL > CCP > MF > MR > PE > MGL (Fig. 1C). The WSR of the MGL was significantly lower than that of the MIX (11.85) and CCP (10.72; Fig. 1C). In addition, the WSRs of the PE (7.30) and MRS (8.03) were significantly lower than that of the MIX (Fig. 1C). Although there was no significant difference between the MGL and the MIX in terms of the numbers of workers and soldiers, the mean number of workers receiving MGL nutrition (579.00) decreased compared to that of the MIX (1038.67), while the mean numbers of soldiers receiving MGL and MIX (89.33 and 90.00, respectively) were very close (Fig. 1D and E).

### Physiological and biochemical states of *O. formosanus* under different nutritional conditions

**Fig. 2:**
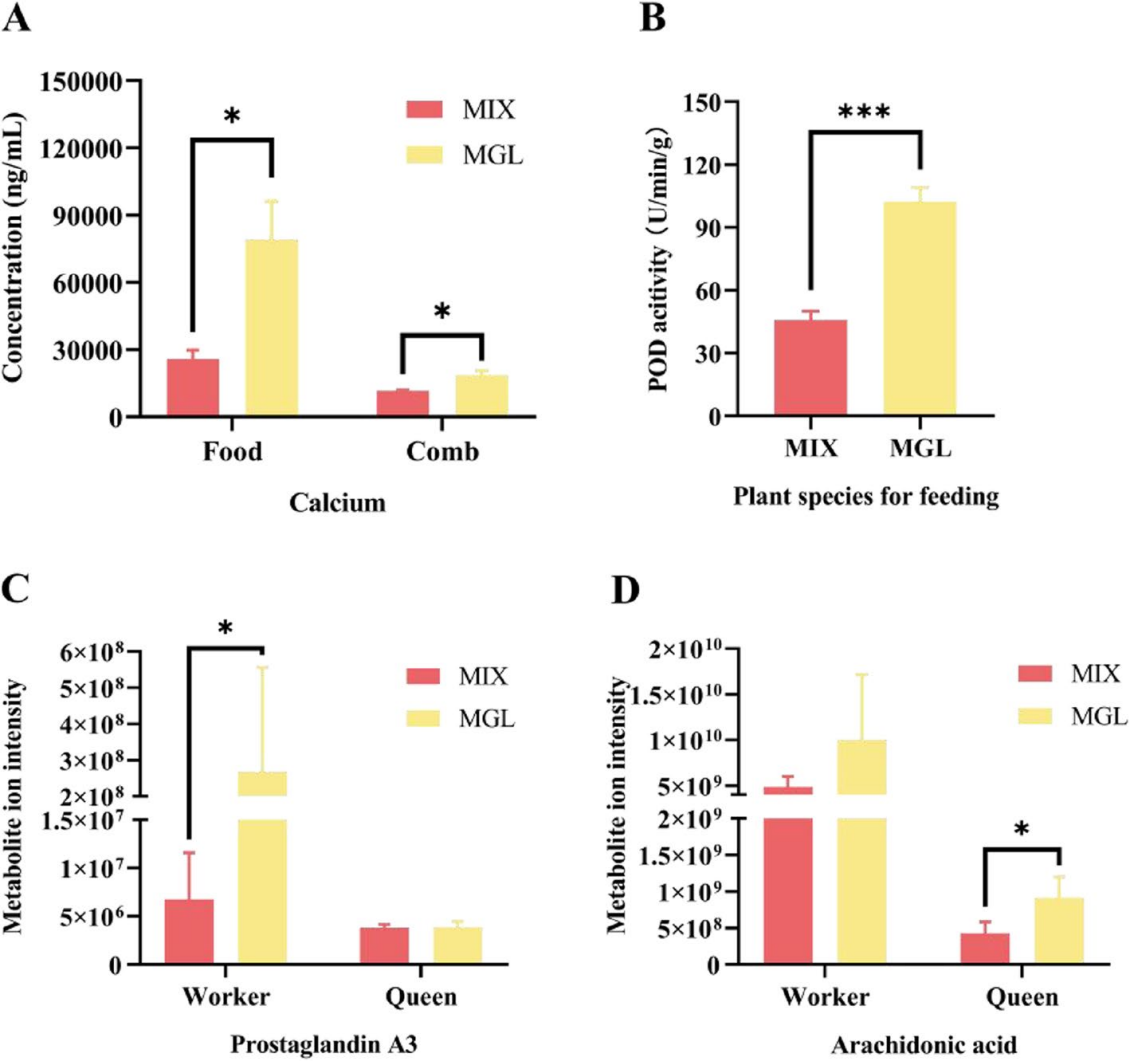
Physiological states of *O. formosanus* under MGL nutrition. Asterisks above the bars indicate significant differences between the treatment and the control (one asterisk indicates *P* < 0.05; two asterisks indicate *P* < 0.01; three asterisks indicate *P* < 0.001; independent samples test). **A** Calcium concentration in foods and combs under MGL nutrition. **B** POD activity in workers receiving MGL nutrition. **C** Metabolite ion intensity of PGA_3_ in workers and queens under MGL nutrition. **D** Metabolite ion intensity of AA in workers and queens under MGL nutrition

The results of ICP-MS analysis of 14 elements in foods and combs of the MGL and MIX showed that the concentrations of Ca and Mg in the MGL were significantly higher than those in the MIX, while the concentrations of Ca, Al, Fe, Zn, Ba, Pb, Ni, V, and Co in combs under MGL nutrition were significantly greater than those in the MIX (Online Resources 1 and 2). The concentration of Cu in combs under MGL nutrition was significantly lower than that under MIX nutrition (Online resource 2). Notably, the concentrations of Ca in the foods and combs under MGL nutrition were both significantly higher than those under MIX (Fig. 2A). The results of POD activity tests showed that POD activity in workers was significantly increased under MGL nutrition (8.19 U/min/g) than under MIX nutrition (3.66 U/min/g, detected in the verification experiment; Fig. 2B). The contents of metabolites in queens and workers under MGL and MIX nutrition were detected by LC‒MS/MS. A total of 1614 metabolites were screened in workers and queens (Online Resource 3). Forty-nine metabolites with significant differences were screened in queens, 27 of which were significantly increased and 22 of which were significantly decreased under MGL nutrition (Online Resource 3). A total of 46 metabolites with significant differences were screened in workers, of which 28 metabolites were significantly increased and 18 metabolites were significantly decreased under MGL nutrition (Online Resource 3). A total of 34 pathways were significantly enriched in the queen, and 12 pathways were significantly enriched in the workers according to the KEGG enrichment analysis (Table 2). All significantly enriched metabolic pathways were divided into four groups based on different castes (queen or worker) and differentially abundant metabolites (positive ions or negative ions), and the top 20 most significant metabolic pathways in each group are shown in the form of a bubble diagram (Online Resources 4, 5, 6, and 7). Among the 46 enriched pathways, "necroptosis" was the most significant, and its corresponding differentially abundant metabolite was AA (Table 2, Online Resource 5). Considering the role of AA in insect PG synthesis, as well as the role of PGs in apoptosis, we screened all the PGs among all the detected metabolites (Online Resource 8). A total of 10 kinds of PGs were identified from the total metabolites of the queen and workers, and PGA_3_ was the only metabolite whose content significantly differed (Online Resource 8). Figure 2C and D show that PGA_3_ and its precursor AA were significantly up-regulated under MGL nutrition in workers and the queen, respectively. Considering the role of PGs in insect reproduction and development, PGA3 may be the key to the inhibitory effect of MGL nutrition on colony development in termites.

**Table 2:** KEGG pathway enrichment results of differentially abundant metabolites in queens and workers under MGL nutrition

Compared groups	Map title	*P* value	Corresponding metabolites
MGL-Q vs.MIX-Q	Necroptosis	0.020	Arachidonic acid
	Fc gamma R-mediated phagocytosis	0.020	Arachidonic acid
	GnRH signaling pathway	0.020	Arachidonic acid
	Ovarian steroidogenesis	0.020	Arachidonic acid
	Linoleic acid metabolism	0.040	Arachidonic acid
	Long-term depression	0.040	Arachidonic acid
	Phototransduction—fly	0.040	Arachidonic acid
	Fc epsilon RI signaling pathway	0.060	Arachidonic acid
	Retrograde endocannabinoid signaling	0.060	Arachidonic acid
	Inflammatory mediator regulation of TRP channels	0.060	Arachidonic acid
	Oxytocin signaling pathway	0.060	Arachidonic acid
	Fatty acid biosynthesis	0.079	Myristic Acid
	Arachidonic acid metabolism	0.079	Arachidonic acid
	Ferroptosis	0.079	Arachidonic acid
	Vascular smooth muscle contraction	0.079	Arachidonic acid
	Platelet activation	0.079	Arachidonic acid
	Regulation of lipolysis in adipocytes	0.079	Arachidonic acid
	Aldosterone synthesis and secretion	0.079	Arachidonic acid
	Serotonergic synapse	0.098	Arachidonic acid
	Biosynthesis of unsaturated fatty acids	0.117	Arachidonic acid
	Amino sugar and nucleotide sugar metabolism	0.154	N-Acetyl-α-D-glucosamine 1-phosphate
	Metabolic pathways	0.571	N-Acetyl-α-D-glucosamine 1-phosphate; Arachidonic acid; Myristic Acid
	Valine, leucine and isoleucine biosynthesis	0.056	L-Threonine
	Glycine, serine and threonine metabolism	0.083	L-Threonine
	Monobactam biosynthesis	0.083	L-Threonine
	Porphyrin and chlorophyll metabolism	0.083	L-Threonine
	Drug metabolism—cytochrome P450	0.083	10-Hydroxycarbazepine
	Mineral absorption	0.209	L-Threonine
	Glutathione metabolism	0.135	Pyroglutamic acid
	Nicotinate and nicotinamide metabolism	0.135	Trigonelline
	Aminoacyl-tRNA biosynthesis	0.160	L-Threonine
	Protein digestion and absorption	0.209	L-Threonine
	Biosynthesis of amino acids	0.233	L-Threonine
	ABC transporters	0.233	L-Threonine
MGL-W vs.MIX-W	Steroid hormone biosynthesis	0.053	Adrenosterone
	Purine metabolism	0.155	Deoxyinosine
	Phosphatidylinositol signaling system	0.035	Inositol
	Ascorbate and aldarate metabolism	0.069	Inositol
	Inositol phosphate metabolism	0.069	Inositol
	Thyroid hormone synthesis	0.069	3-Iodo-L-tyrosine
	Steroid hormone biosynthesis	0.076	Deoxycorticosterone; Etiocholanolone
	Galactose metabolism	0.135	Inositol
	Aldosterone synthesis and secretion	0.134936975	Deoxycorticosterone
	ABC transporters	0.283	Inositol
	Neuroactive ligand‒receptor interaction	0.283	N-Oleoyl dopamine
	Tyrosine metabolism	0.336	3-Iodo-L-tyrosine

### Effects of PGA_3_ and AA on the comb and population development of *O. formosanus*

**Fig. 3:**
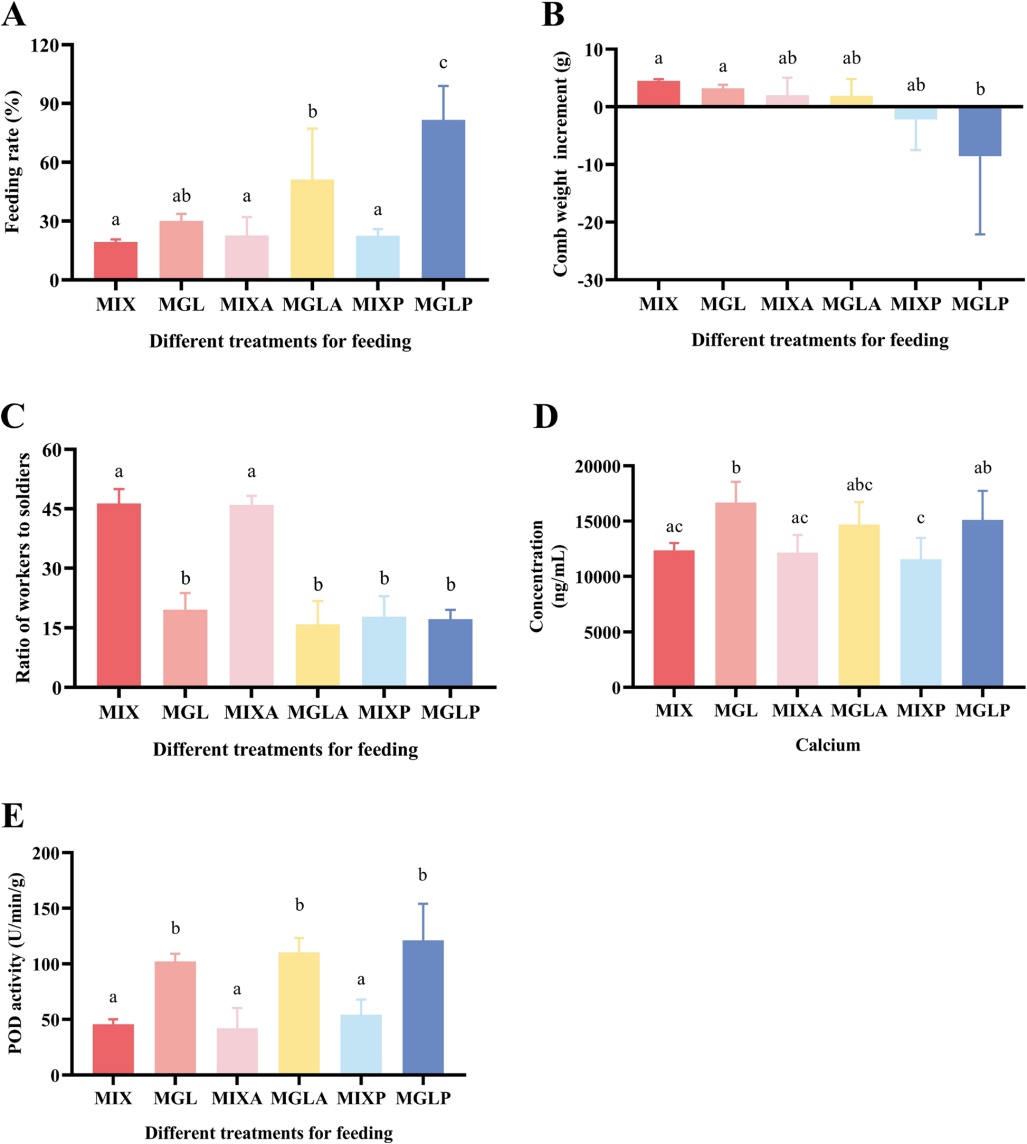
Population development and physiological status of *O. formosanus* after AA and PGA_3_ feeding. Different letters above the bars indicate different statistical groups. Two sets of letters that do not overlap indicate a significant difference between the two corresponding treatments; there is no significant difference between the two treatments that are included in any of the same statistical groups (*P* < 0.05, one-way ANOVA). **A** Feeding rates of termites supplied with AA and PGA_3_. **B** Comb weight increases under AA and PGA_3_ nutrition. **C** Calcium concentration in combs under AA and PGA_3_ nutrition. **E** POD activity in workers receiving AA and PGA_3_. **D** Ratio of workers to soldiers receiving AA and PGA_3_

To further investigate the effects of AA and PGA_3_ on the colony development of *O. formosanus*, a verification experiment was conducted. The results showed that the feeding preference of workers for MGL was not influenced by the addition of chemicals (Fig. 3A). All the MGL groups (MGL, MGLA, and MGLP) had higher FRs than did all the MIX groups (MIX, MIXA, and MIXP; Fig. 3A). The FRs of the MGLA and MGLP groups were significantly higher than those of all the MIX groups, while the FR of the MGLP group was significantly greater than that of all the other five groups, reaching 81.64% (Fig. 3A). However, the CWI was negative under MGLP nutrition (-8.60 g) and was significantly lower than that under MIX and MGL (4.50 g and 3.20 g, respectively; Fig. 3B). There was no significant difference between the MGL and MIX in the CWI, probably due to the shortened culture time (Fig. 3B). The WSRs of all the MGL groups were significantly lower than those of the MIX and MIXA groups (Fig. 3C). This finding was consistent with the effect of MGL on the WSR in the feeding experiment and further confirmed the inhibitory effect of MGL on the development of workers (Fig. 1C). Notably, the WSR of the MIXP (17.87) was significantly lower than that of the MIX and MIXA (46.32 and 45.97, respectively; Fig. 3C). Although there was no significant difference between the CWI of the MIXP and that of the MIX and MIXA, it showed negative growth in the MIXP (-2.17 g), which was similar to that of the MGLP (Fig. 3B). These results suggested that the MIX combined with PGA_3_ had an inhibitory effect on comb and worker development, similar to what was observed for MGL and MGLP (Fig. 3B and C). The contradiction between FR and colony development under MGLP nutrition was similar to that of MGL in the feeding experiment (Figs. 1A, B, 3A, and B). These results strongly demonstrated the inhibitory effect of PGA_3_ on the comb and worker development of *O. formosanus*.

In addition, the concentration of calcium in the combs and POD activity in the workers were also detected. The concentrations of Ca in all the MGL groups were greater than those in all the MIX groups (Fig. 3D). The POD activities of the MGL, MGLA and MGLP (8.19 U/min/g, 8.84 U/min/g and 9.69 U/min/g, respectively) were significantly greater than those of the MIX, MIXA and MIXP (3.66 U/min/g, 3.36 U/min/g and 4.35 U/min/g, respectively; Fig. 3E). These results were highly consistent with the results of the feeding experiment. (Figs. 2A, B, 3D and E).

## Discussion

**Fig. 4:**
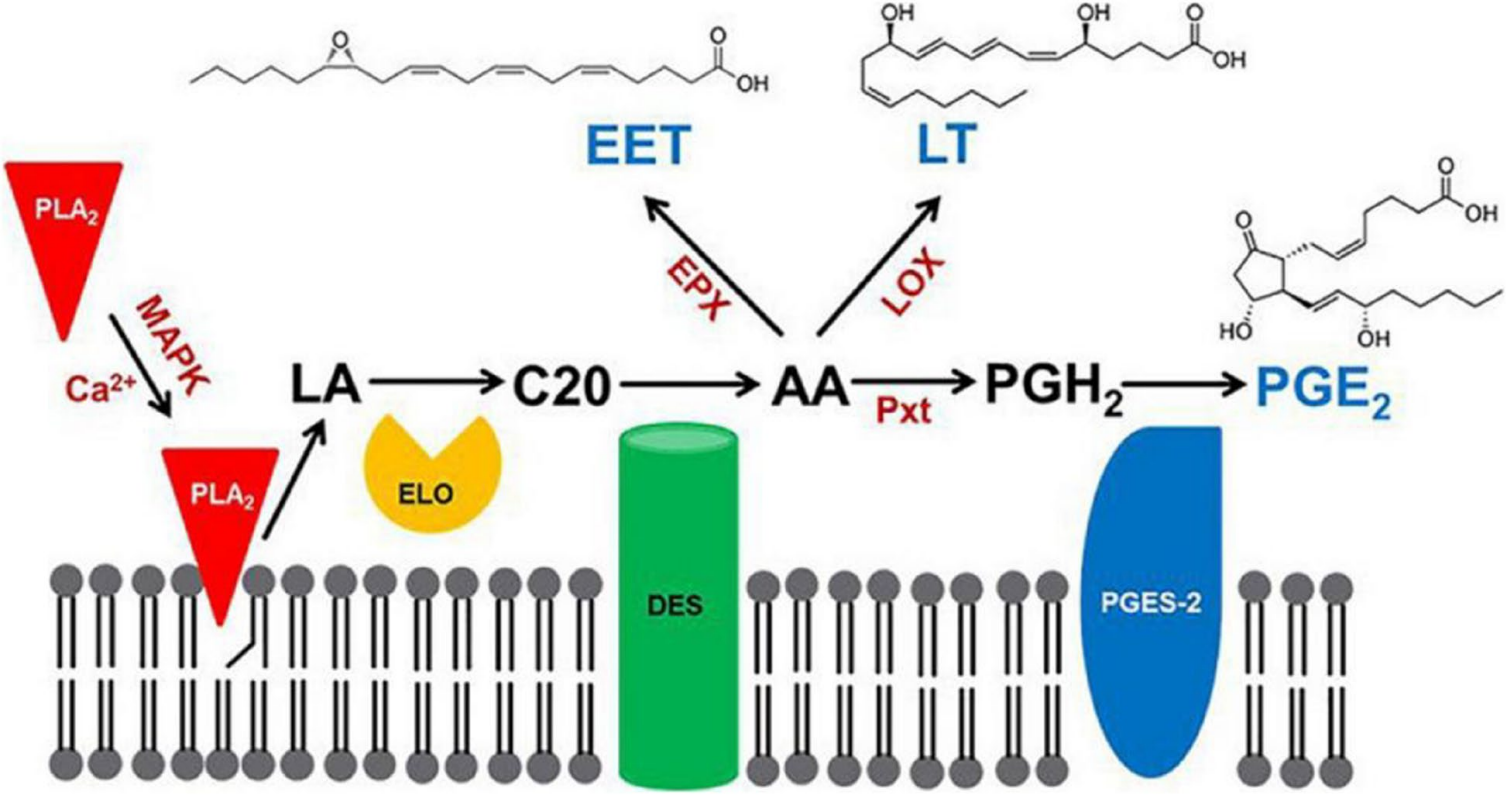
A model for eicosanoid biosynthesis in insects (modified from Stanley and Kim [46]). Insect phospholipase A2 (PLA_2_) is released from biofilms under the activation of Ca.^2+^ or mitogen-activated protein kinase (MAPK), which catalyzes the hydrolysis of linoleic acid (LA), extending it to C20 fatty acid (C20) under the action of a specific elongase (ELO). C20 is oxidized to arachidonic acid (AA) by desaturase (DES). AA is oxygenated to epoxyeicosatrienoic acid (EET) by epoxidase (EPX), to leukotriene (LT) by lipoxygenase (LOX), and to prostaglandin H2 (PGH_2)_ by peroxidase (peroxinectin: Pxt). PGH_2_ is isomerized to PGE_2_ through the action of PGE_2_ synthase-2 (PGES-2) [46]

AA (20:4n-6) is a polyunsaturated fatty acid (PUFA) that is a precursor of a series of eicosanoids, including PGs [33]. PGs are a group of signaling molecules involved in reproduction, inflammation, immunity, organ formation, apoptosis, body temperature regulation, and blood pressure regulation that mediate a variety of physiological and pathological responses in both vertebrates and invertebrates [34–36]. Although the amount of PGs synthesized in insects is much lower than that in mammals, PGs are involved in a wide range of physiological activities, such as follicle development, egg laying, larval development, and Malpighian tubule physiology, in more than twenty insect species, including crickets, cockroaches, mosquitoes, silk moths, locusts, and bugs [35, 37–45]. Figure 4 [46] shows the general synthesis process of PGs (taking PGE_2_ as an example) in insects. Insect phospholipase A_2_ (PLA_2_) is released from the membrane under the activation of Ca^2+^ or mitogen-activated protein kinase (MAPK) [37]. PLA_2_ and a specific elongase (ELO) catalyze the hydrolysis and extension of linoleic acid (LA), respectively, converting it to a C20 fatty acid (C20) [46]. C20 is oxidized to AA by desaturase (DES) [46]. AA is oxygenated to PGH_2_ via POD (peroxinectin: Pxt) [37, 46, 47]. PGH_2_ is ultimately isomerized into a variety of PGs (PGD_2_, PGE_2_, PGF_2α_, PGI_2_, and TXA_2_) [48]. However, the biosynthetic route and biological functions of PGAs are still unclear. A potential synthetic route for PGAs is to convert AA into hydroxylated allene oxide and form PGAs through cyclization [49].

The role of PGs in termites remains unknown, and there is  little research on the function of PGAs in living organisms. In this study, we investigated a series of ecological and physiological performances of *O. formosanus* under MGL nutrition and confirmed the role of PGA_3_ on the population development regulation of *O. formosanus*. CWI and WSR are considered to be important indicators of the development status of a termite colony [46, 50]. The WSR in a particular termite species is usually maintained at a relatively stable value during a particular stage of development [51, 52]. In a fungus-growing termite colony, workers are almost responsible for all of the work except reproduction in the process of population development and comb establishment, while soldiers not only do not have the ability to forage, but also need feeding and grooming [53–58]. Therefore, maintaining a high proportion of soldiers in a colony is costly, and ensuring the proportion of workers is important [59]. It was found that increasing the proportion of soldiers on the basis of the original WSR would significantly reduce the survival rate of colonies of *Coptotermes formosanus* [60]. Due to the difference of energy consumption mechanism between workers and soldiers, the colony nutritional status influence the WSR a lot. Termites may lose the ability to continuously differentiate soldiers under insufficient nutrient intake [61]. Correspondingly, the change of the WSR has a great impact on the colony development [57, 59, 60, 62]. Controlling the WSR in a reasonable range and achieving a balance between productivity and defense is crucial [46, 52, 63, 64]. In addition to the nutritional status of the colony, the WSR is also affected by species, region, age, and season (higher in winter) [46, 52, 63, 64]. All experiments in this study used colonies of around one year old collected from the same area at the same time, making the nutrients the only variable. The feeding experiment showed that the overall development of colonies fed with multiple nutrients was better than those cultivated under single nutrition. Colonies under MGL nutrition had the highest FR, the lowest CWI, and the lowest WSR among all the seven groups, while the ranking of the other six groups changed little among the three indicators. These results supposed that MGL nutrition adversely impacted the colony development. The lowest WSR indicates that the poor growth status under MGL nutrition may not be caused by energy deficiency, but by stunted development of workers, which is consistent with the highest FR [61].

The adverse effects of MGL nutrition probably started from an excessive intake of calcium by workers. The ICP-MS analysis showed that the concentration of calcium in MGL was significantly higher than that in the MIX. A higher concentration of calcium was also detected in combs under all MGL nutrition. Calcium is an essential signal molecule in the metabolism of almost all organisms, including insects, and is involved in the biosynthesis of lipids, proteins, and carbohydrates, and countless enzymatic reactions [46, 65–69]. Calcium in the comb is circulating within the termite colony [58, 70–72]. The comb is fermented from the feces of workers, and the calcium content in comb directly reflects the inner environment of the workers' guts [58]. The mature comb is eaten by workers, and the *Termitomyces* nodules growing on the comb are eaten by the reproductive castes [58, 70–72]. The queen also exchanges nutrients with workers and controls worker development through trophallaxis and pheromones [63]. Therefore, the dynamic change of calcium concentration in the comb has a significant impact on the metabolism of the colony. The high concentration of calcium in combs under MGL nutrition stimulated a series of metabolic activities, including the biosynthesis of LA [46]. Termites are counted among the organisms known capable to synthesize LA de novo [46, 73–75]. Lack of exogenous intake results in a low LA content in termites [73]. The synthesized LA was converted into AA or other PUFAs immediately [73]. These factors  likely led to a significant up-regulation of AA in queen under MGL nutrition.

PODs are involved in multiple physiological activities varying in immunity, detoxification, oxidation of fatty acids, regulation of oxygen concentration, and metabolism of nitrogenous chemicals [76]. The conversion from AA to PGs in insects is also catalyzed by a POD (Pxt) [46]. In this study, we found that all MGL nutrition (MGL, MGLA, and MGLP) significantly increased POD activity in workers.

The up-regulation of calcium content in the comb, the enhancement of POD activity in workers, and the up-regulation of AA in the queen eventually led to the up-regulation of PGA_3_ in workers [77]. Based on the roles of PGs in insects, we  hypothesized that MGL's inhibition to the colony development of *O. formosanus* was  likely due to the excessive synthesis of PGA_3_.

The results of the verification experiment strongly supported the hypothesis above. Colonies fed by the MIXP showed similar performances (negative CWI and significantly lower WSR) to those fed by MGL, while there were no significant differences between the MIXA and the MIX in all indicators. The addition of AA and PGA_3_ had no significant impacts on the FR, calcium concentration in combs, and POD activity in workers. These results indicated that the inhibitory effect of MGL nutrition was dominated by PGA_3_, while Ca^2+^, POD, and AA were auxiliary factors. The significant decrease in WSR is consistent with the results in the feeding experiment, which indicated that PGA_3_ probably regulated the colony development by inhibiting the development of workers. In addition, similar to other PGs, the regulation of PGA_3_ is likely to be dose-related, as the colony development states under MGLP nutrition were worse than those under MGL nutrition [37].

Wang et al. [35] suggested that PGA_2_ induced apoptosis in three cell lines derived from the fall armyworm, *Spodoptera frugiperda* in a dose-related way, proving that the regulation of PGs on apoptosis is not limited to mammals. Here, the KEGG enrichment analysis showed that the necroptosis involved by AA was significantly up-regulated in the queen under MGL nutrition. This study provides new evidence for the possible role of AA and PGAs in insect apoptosis.

Our study showed that termites, like some of their cockroach ancestors, may be able to oxidize AA to synthesize PGs and that PGA_3_ plays a role in the regulation of colony development of *O. formosanus* by reducing the worker proportion [46]. Stunted worker development left the entire colony unhealthy, as workers were responsible for the vital tasks of foraging, tunneling, caring for eggs, raising larvae and soldiers, and building combs [63]. MGL is a common landscape plant widely distributed in the distribution area of *O. formosanus* in China, and PGs can be metabolized by most known organisms, including plants and mammals [27, 46, 78, 79]. Considering the significantly higher FRs under MGL and MGLP nutrition, the probably new bait made of MGL and PGA_3_  shows promise as a sustainable and effective termite control method. This study provides a new idea for the development of environmentally friendly biological control methods for higher subterranean termites, and reveals the potential function of PGs in termite development for the first time.

## Supplementary Information

Supplementary Material 1.

Supplementary Material 2.

Supplementary Material 3.

Supplementary Material 4.

Supplementary Material 5.

Supplementary Material 6.

Supplementary Material 7.

Supplementary Material 8.

## Data Availability

The data that support the findings of this study are openly available.
