# Correction: Prostaglandin A3 regulates the colony development of *Odontotermes formosanus* by reducing worker proportion

**DOI:** 10.1007/s44297-025-00043-6

**Published:** 2025-01-16

**Authors:** Qihuan Zhou, Ting Yu, Wuhan Li, Raghda Nasser, Nooney Chidwala, Jianchu Mo

**Affiliations:** 1 Ministry of Agriculture Key Lab of Molecular Biology of Crop Pathogens and Insect Pests, Key Laboratory of Biology of Crop Pathogens and Insects of Zhejiang Province, Institute of Insect Sciences, College of Agriculture and Biotechnology, Zhejiang University, Hangzhou, 310058 China; 2 Department of Zoology and Entomology, Faculty of Science, Minia University, El-Minia, 61519 Egypt

**Correction: Crop Health 2, 11 (2024)**

**https://doi.org/10.1007/s44297-024–00030-3**

Following publication of the original article [1], the authors reported that the Acknowledgements and Funding sections need to be revised.

The Acknowledgements section is corrected from:

**Acknowledgements**

The authors would like to thank the financial support provided through the National Natural Science Foundation of China (No. 32071771) and the European Research Council Consolidator (No. ERC-CoG 771349).

To:

**Acknowledgements**

The authors would like to thank the financial support provided through the National Natural Science Foundation of China (No. 32071771).

The Funding section is corrected from:

**Funding**

This research was supported by the National Natural Science Foundation of China (No. 32071771); The European Research Council Consolidator (No. ERC-CoG 771349).

To:

**Funding**

This research was supported by the National Natural Science Foundation of China (No. 32071771).

The original article [1] has been updated.
