# Supplementary material for: Prostaglandin A3 regulates the colony development of Odontotermes formosanus by reducing worker proportion
Source: Crop Health. 2024 Jul 2;2(1):11. doi: 10.1007/s44297-024-00030-3 (PMC11232360; doi:10.1007/s44297-024-00030-3)
Supplement: Supplementary file 1 — Supplementary Material 1. [file 44297_2024_30_MOESM1_ESM.zip › Online Resource 1.pdf]

Prostaglandin A3 regulates the colony development of *Odontotermes formosanus* by reducing worker proportion

Crop health

Qihuan Zhou[1], Ting Yu[1], Wuhan Li[1], Raghda Nasser[1, 2], Nooney Chidwala[1], Jianchu Mo[1]*

Online Resource 1 Content of 14 elements in MGL and MIX

Elements	MIX (ng/mL)	MGL (ng/mL)	P value
Ca	25936.72±2244.88	79201.38±9825.30	0.027
Al	826.39±290.44	463.60±54.37	0.337
Mn	69.79±6.69	64.73±4.86	0.574
Fe	1033.69±6.69	802.99±4.86	0.574
Zn	199.10±42.81	501.41±380.10	0.51
Na	568.32±144.76	430.30±11.28	0.441
Mg	1369.60±70.33	2567.45±130.50	0.001
Ba	117.52±56.11	39.93±4.43	0.301
Cu	16.33±1.77	15.16±1.67	0.655
Pb	6.51±1.56	8.76±1.42	0.316
Ni	3.63±1.13	3.00±0.41	0.623
Cr	16.56±2.72	14.31±2.98	0.607
V	1.48±0.24	1.37±0.18	0.731
Co	1.13±0.25	0.73±0.12	0.221
