# Supplementary material for: Prostaglandin A3 regulates the colony development of Odontotermes formosanus by reducing worker proportion
Source: Crop Health. 2024 Jul 2;2(1):11. doi: 10.1007/s44297-024-00030-3 (PMC11232360; doi:10.1007/s44297-024-00030-3)
Supplement: Supplementary file 2 — Supplementary Material 2. [file 44297_2024_30_MOESM2_ESM.zip › Online Resource 2.pdf]

Prostaglandin A3 regulates the colony development of *Odontotermes formosanus* by reducing worker proportion

Crop health

Qihuan Zhou[1], Ting Yu[1], Wuhan Li[1], Raghda Nasser[1, 2], Nooney Chidwala[1], Jianchu Mo[1]*

Online Resource 2 Content of 14 elements in combs under the MIX or MGL nutrition

Elements	MIX (ng/mL)	MGL (ng/mL)	P value
Ca	11632.21±216.24	18554.23±1202.57	0.026
Al	707.64±116.99	3272.81±270.11	0.001
Mn	621.18±26.66	664.42±50.08	0.488
Fe	475.15±46.54	926.77±92.38	0.012
Zn	105.38±9.46	184.12±24.07	0.038
Na	1671.55±44.88	1950.89±168.69	0.185
Mg	3556.21±196.54	3797.55±228.29	0.468
Ba	63.70±3.74	107.11±4.63	0.002
Cu	153.10±23.59	80.88±3.10	0.039
Pb	12.64±3.35	24.62±1.22	0.028
Ni	5.26±0.49	13.41±2.61	0.037
Cr	8.19±0.63	13.16±2.46	0.122
V	2.99±0.26	5.54±0.46	0.009
Co	0.72±0.06	2.36±0.18	0.001
