# Supplementary material for: Prostaglandin A3 regulates the colony development of Odontotermes formosanus by reducing worker proportion
Source: Crop Health. 2024 Jul 2;2(1):11. doi: 10.1007/s44297-024-00030-3 (PMC11232360; doi:10.1007/s44297-024-00030-3)
Supplement: Supplementary file 3 — Supplementary Material 3. [file 44297_2024_30_MOESM3_ESM.zip › Online Resource 3.pdf]

Prostaglandin A3 regulates the colony development of *Odontotermes formosanus* by reducing worker proportion

Crop health

Qihuan Zhou[1], Ting Yu[1], Wuhan Li[1], Raghda Nasser1, [2], Nooney Chidwala[1], Jianchu Mo[1]*

Online Resource 3 Differential metabolites in workers and queen under the MGL and MIX nutrition

Compared Samples	The Number of Total Identification	The Number of Total Identification	The Number of Significantly Up	The Number of Significantly Down
MGL-Q vs MIX-Q	1614	49	26	22
MGL-W vs MIX-W	1614	46	29	18
