# Supplementary material for: Prostaglandin A3 regulates the colony development of Odontotermes formosanus by reducing worker proportion
Source: Crop Health. 2024 Jul 2;2(1):11. doi: 10.1007/s44297-024-00030-3 (PMC11232360; doi:10.1007/s44297-024-00030-3)
Supplement: Supplementary file 6 — Supplementary Material 6. [file 44297_2024_30_MOESM6_ESM.pdf]

Prostaglandin A3 regulates the colony development of *Odontotermes formosanus* by reducing worker proportion

Qihuan Zhou[1], Ting Yu[1], Wuhan Li[1], Raghda Nasser[1, 2], Nooney Chidwala[1], Jianchu Mo[1]*

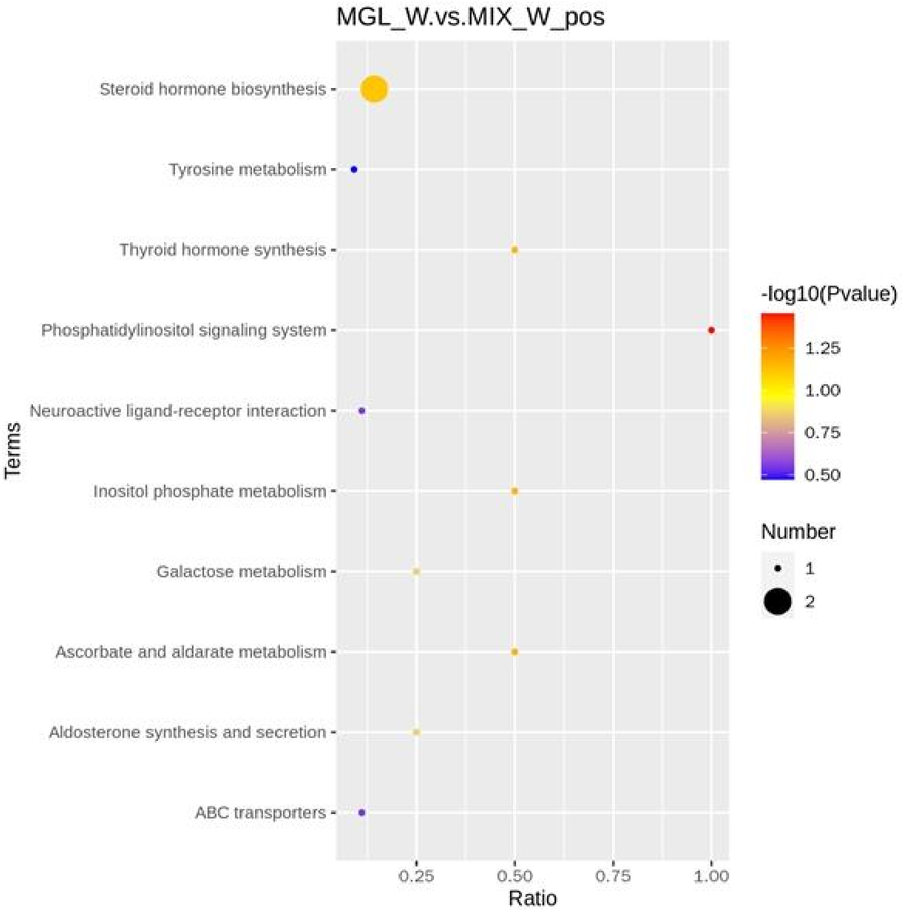

Online Resource 6 KEGG enrichment results of positive differential metabolites in workers under the MIX and MGL nutrition (Top 20). The horizontal coordinate in the figure is x/y (the number of differentiated metabolites in the corresponding metabolic pathway/the total number of identified metabolites in the pathway. A higher value indicates a higher concentration of differentiated metabolites in the pathway. The color of the dots represents the p-value. The size of the dot corresponds to the number of differentiated metabolites in the corresponding pathway, with larger dots indicating more differentiated metabolites in the pathway
