# Supplementary material for: Prostaglandin A3 regulates the colony development of Odontotermes formosanus by reducing worker proportion
Source: Crop Health. 2024 Jul 2;2(1):11. doi: 10.1007/s44297-024-00030-3 (PMC11232360; doi:10.1007/s44297-024-00030-3)
Supplement: Supplementary file 8 — Supplementary Material 8. [file 44297_2024_30_MOESM8_ESM.zip › online resource 8.pdf]

Online resource 8 The difference of PGs contents in workers and queens under the MGL nutrition

Metabolite name	Difference indexes of contents in workers				Difference indexes of contents in queens			
	log2FC	P value	VIP	Up/down	log2FC	P value	VIP	Up/down
$PGF_{3α}$	-0.03366703	0.883365255	0.212677579	Down	-0.176516213	0.860247739	0.394442007	Down
PGH_1	-1.32528721	0.370843793	1.016271875	Down	-0.343511839	0.302895278	0.322554591	Down
PGG_2	-2.035500575	0.704256975	0.759540321	Down	-0.065856256	0.774446914	0.079382388	Down
PGD_2	-1.73769749	0.171268961	1.388842485	Down	0.07428254	0.745075884	0.060590113	Up
PGH_2	0.963323596	0.459972425	0.755485134	Up	0.72309427	0.840641025	0.333230261	Up
PGD_1	-1.665674794	0.494692243	0.822467767	Down	-0.359071245	0.11263455	0.266632499	Down
PGA_3	-5.304636159	0.015395519	3.105711688	Up	0.011628169	0.984035847	0.002990696	Up
PGK_1	0.617176827	0.028348237	0.596539299	Up	0.942658301	0.482655308	0.772066876	Up
PGK_2	-2.037889468	0.435086245	1.211574871	Down	0.140466797	0.769159361	0.18976882	Up
PGJ_2	0.219620299	0.515019066	0.30768169	Up	0.436139617	0.27835992	0.341799818	Up
